# Mesoporous silica-based nanoplatforms for the delivery of photodynamic therapy agents

**DOI:** 10.1007/s40005-017-0356-2

**Published:** 2017-09-09

**Authors:** Suk ho Hong, Yongdoo Choi

**Affiliations:** Biomarker Branch, National Cancer Center, 323 Ilsan-ro, Goyang-si, Gyeonggi-do 10408 Republic of Korea

**Keywords:** Photodynamic therapy, Mesoporous silica, Drug delivery, Nanocarrier, Biocompatibility, Theranostic platform, Controlled release

## Abstract

Photodynamic therapy (PDT) is an established method for the treatment of cancer which utilizes light, a photosensitizer (PS), and oxygen. Unfavourable characteristics of most PSs, such as low solubility and tumour specificity have led many researchers to adopt nanoscale drug delivery platforms for use in PDT. Mesoporous silica nanoparticles (MSNs) form a significant part of that effort, due to their ease and controllability of synthesis, ease of loading, availability of diverse surface functionalization, and biocompatibility. Therefore, in this review, we discuss the properties of MSNs as they pertain to their use in PDT and review the latest advances in the field, comparing the different approaches currently being used.

## Introduction

Photodynamic therapy (PDT) is a clinically approved, and minimally invasive treatment modality, that involves the combination of individually non-toxic components of light, photosensitizer (PS) and oxygen (Dolmans et al. 2003). When the PS is illuminated with a specific wavelength of light, the PS molecules transfer their energy to neighbouring tissue oxygen molecules, thereby generating reactive oxygen species (ROS), including singlet oxygen and free radicals. This resulting ROS is highly cytotoxic, and thus PDT can be, and has been, successfully used to treat various types of cancers and non-malignant diseases (Dolmans et al. 2003). Because PS molecules do not have significant toxicity until activated by light input, systemic side effects can be avoided (Dolmans et al. 2003; Lucky et al. 2015). Thus, this method displays far less collateral damage than other conventional cancer therapy methods.

Regardless of the potential benefits, the properties of PDT agents have limited their application in clinical use. The reasons for this are numerous. First, most PSs are hydrophobic, and are either relatively insoluble in water, or will aggregate in aqueous solutions, resulting in altered properties including their singlet oxygen quantum yield (Bechet et al. 2008; Couleaud et al. 2010). Second, PDT agents often have an unfavourable bio-distribution, including a high accumulation in the skin that can lead to prolonged skin sensitivity (Konan et al. 2002). Although this issue is somewhat mitigated by the fact that irradiation is required to generate toxicity, it does highlight another shortcoming for PDT, namely that light cannot typically penetrate a tissue beyond 3 mm because of high tissue absorption (Lucky et al. 2015). Finally, PDT agents tend to have poor selectivity for tumour tissue over normal tissues. In light of these issues, researchers have sought to create delivery systems that can effectively incorporate PDT agents and make PDT a more viable approach (Konan et al. 2002).

Nanomaterials have been used in drug delivery for some time now, and PDT agents have been no exception to this, since nanomaterials allow PS drugs to overcome their limitations (Lucky et al. 2015). By encapsulating PS molecules in nanostructures, the hydrophobicity of each PS can be disregarded; the resulting complex can readily be made to be hydrophilic (Bechet et al. 2008). In addition, through surface modification of the nanostructures with targeting moieties such as peptides, antibodies, and aptamers, PDT agents can be directed to target cells with high specificity (Lucky et al. 2015). Even without such measures, nanoparticles can take advantage of a tumour targeting effect due to enhanced permeability and retention (EPR) of tumour tissues (Couleaud et al. 2010; Hashizume et al. 2000; Lucky et al. 2015).

While a diverse selection of nanoparticles (NPs) has been used to develop a platform for PS delivery, including polymers, lipids, metals and other inorganic materials, (Bechet et al. 2008; Kwon et al. 2013; Li et al. 2012; Lucky et al. 2015) mesoporous silica has been shown to have several advantages as a PS carrier (Couleaud et al. 2010; Kwon et al. 2013). Mesoporous silica nanoparticles (MSNs) are a family of porous silica NPs that have pore sizes ranging from 2 to 50 nm. Since the 1990s, a variety of structures utilizing supra-molecularly arranged surfactant as templates have emerged (Bharti et al. 2015); the more widely-known examples include MCM-41 and SBA-15. The key strengths of MSNs are as follows: their porosity and consequent large surface area provide an optimal setting for loading of drugs by adsorption; the ability to control pore size and dimensions allows for control of the drug release dynamics; the ease of surface modification allows for targeting, imaging and other auxiliary functions to be incorporated; their biocompatibility, and the extensive knowledge available around their degradation and excretion (Vivero-Escoto et al. 2010). Hence, MSNs have been widely employed as carriers for PS drugs. Here we discuss the biocompatibility of MSNs, then outline recent advances and developments in the use of MSNs as a delivery system for PDT agents.

## Principles of photodynamic therapy

PDT generally proceeds as follows (Dolmans et al. 2003): first, the PS absorbs a photon of light and moves from the ground state to an excited singlet state. The excited PS then can return to the ground state, emitting fluorescence in the process; this allows for the use of PSs in fluorescent imaging and sensing applications. Alternatively, the PS can undergo intersystem crossing to become a longer-lived triplet excited state. In this state, the PS can transfer its charge to another molecule to produce a free radical, which in turn will react with oxygen to make oxygenic species, including hydroxyl and superoxide radicals (called a Type I transfer). If the PS transfers the energy directly to molecular oxygen, the oxygen will go from the triplet ground state to a singlet excited state (called a Type II transfer). Both these reactions produce what serve as the effective components of PDT. However, most studies use singlet oxygen generation (SOG) as representative of the approach (Lucky et al. 2015). Since singlet oxygen is short-lived, the tissue damage location is equivalent to that of the PS itself (Ormond and Freeman 2013). In tumour cells, oxidation of plasma membranes or organelles such as mitochondria, the Golgi apparatus, and the endoplasmic reticulum induces irreversible damage, and leads to cell death by apoptosis, necrosis, or autophagy (Lucky et al. 2015).

## Mesoporous silica nanoparticles

Silica based nanoparticles have been researched extensively because of their advantages including chemical stability and tenability (Couleaud et al. 2010). While a variety of silica materials exist, including Stöber silica (solid/dense) and organically modified silica, mesoporous silica is characterized by the presence of mesopores, which provide the material with a large surface area (Bharti et al. 2015). As outlined in the previous section, MSNs are a prominent PS delivery system that confers numerous advantages. MSN delivery systems are differentiated by a number of factors including pore geometry, pore size, and drug incorporation methods, which in turn affects the drug loading and release characteristics (Kwon et al. 2013).

Although there are a number of synthesis methods used for the production of MSNs, typically a sol–gel process is used (Couleaud et al. 2010; Kwon et al. 2013; Li et al. 2012). This is a simple method where hydrolysis and condensation of organosilane precursors, such as tetraethyl orthosilicate (TEOS), produce colloids of silica particles. The reaction is performed in the presence of micelles formed by surfactants, so that silica will form a structure based on the micelle template. The most widespread pore type is MCM-41, a structure of cylindrical mesopores arranged in a hexagonal order (Bharti et al. 2015; Kwon et al. 2013; Tarn et al. 2013). It is mainly synthesized using cetyltrimethylammonium bromide (CTAB) as a template, and has pore sizes of 2–10 nm. Other notable structures include SBA-15, another two-dimensional hexagonal structure created using Pluronic P123 as the template, and MCM-48, a three-dimensional cubic structure (Beltrán-Osuna and Perilla 2016).

Alternative forms of mesoporous silica have also been extensively explored, the most important of which are hollow and rattle types. A hollow/rattle-like MSN is created using one of the following strategies: using a soft template in synthesis; using a hard template that can be removed later; by surface-protecting the nanoparticle and then etching the core out; and lastly by creating a complex structure from the bottom-up without templates. Because of their improved density, surface area, and drug loading capacity, these structures are regarded as being the next-generation MSNs (Bharti et al. 2015; Shi et al. 2013).

## Biocompatibility of mesoporous silica nanoparticles

One of major concerns when devising a drug nanocarrier is the biocompatibility of such a carrier. In the case of silica, the material is known to be, and has accordingly been listed as, ‘generally recognized as safe’ by the U.S. Food and Drug Administration. Since the advent of nanomaterials, it has been noted that their special size and surface area could mean different interaction with cells, and therefore different toxicity compared to silica itself (Li et al. 2015). In the specific case of MSNs, the mesoporosity differentiates the particles further from their dense (amorphous or crystalline) counterparts, and while specific results differ by a number of variables including dosage, particle size and shape, cell lines used and so on, the general consensus is that MSNs have a significantly lower cytotoxicity than dense silica NPs (Asefa and Tao 2012; Tarn et al. 2013).

Extensive assessments on the distribution, translocation, degradation, and excretion of MSNs have been performed. Lu et al. used MSNs fluorescently labelled with FITC to determine their cellular uptake based on their size, ranging from 30 to 280 nm: the data did not show an order with respect to sizes, with an MSN of 50 nm showing the greatest degree of uptake. Importantly, at a dose of 100 μg/mL, no significant cytotoxicity was observed (Lu et al. 2009). Lin and Haynes, acknowledging that there is a disagreement over the size-dependency of MSN cytotoxicity, chose to investigate MSNs less than 100 nm in diameter, because NPs in that range would avoid immediate uptake by the reticuloendothelial system (RES), a property which is desirable for drug delivery. Using MSNs of different sizes, ranging from 25 to 225 nm, they examined haemolysis, and observed that, with the exception of the smallest particle, smaller-sized MSNs tend to be more haemolytic. They also found that MSNs had reduced haemolysis compared to dense silica NPs (Lin and Haynes 2010).

With respect to the shape of the MSN particles, researchers have used various types of cells to determine the effect of shape on MSN uptake by cells. In a study published in 2008, Trewyn et al. compared a spherical MSN, with a diameter of around 100 nm, with rod-shaped MSNs that had a similar width, but with a length of 600 nm. In both Chinese Hamster Ovarian (CHO) cancer cells and non-cancerous human fibroblast cells, the rod shaped particles were taken up more efficiently; while the CHO cells showed faster endocytosis than the fibroblast cells, the latter was especially slow to take up the rod shaped MSN, possibly owing both to the nanorods’ sheer size and their greater tendency to aggregate (Trewyn et al. 2008). Huang et al. used an MSN of 100 nm in diameter (short axis) and aspect ratios (ARs) of 1, 2, and 4 to show that longer particles were more easily internalized by A375 human melanoma cells; the longer particles appeared to disrupt the actin fibres more; cell adhesion was also affected, as the longer particles decreased the expression of melanoma cell adhesion molecule (MCAM), as well as decreasing cell adhesion itself. In addition, although all particles were virtually nontoxic, the longer particles did display somewhat higher cytotoxicity (Huang et al. 2010). One study showed that there was no difference among MSNs with aspect ratios of 1, 2, 4, or 8 with regards to their toxicity (Yu et al. 2011).

Meng et al. showed that an MSN with ARs of 2.1–2.5 were taken up by human cervical cancer (HeLa) cells and human lung adenocarcinoma (A459) cells much better than those with aspect ratios that were lower (i.e. spheres with an AR of 1.5–1.7) or higher (4.0–4.5) (Meng et al. 2011). This can be attributed to the fact that MSNs with ARs of 2.1–2.5 are better able to activate the small GTP binding proteins that lead to macropinocytosis.

It has been suggested that the multitude of energy-dependent internalization pathways and the interplay of various factors of MSN themselves, including size, shape, surface area, and chemistry underscores why so many contradictory results with respect to cellular uptake and cytotoxicity have been reported (Zhao et al. 2011).

Recently, in vivo investigations of MSN bio-distribution have been conducted. In a study published in 2013, Fu et al. exposed mice to a 110 nm nanorattle type MSN using four different methods, namely intravenous injection, hypodermic injection, intramuscular injection, and oral administration. They found that while the MSN was barely absorbed following hypodermic or intramuscular injection, the MSNs were readily absorbed into the system either orally or intravenously. Generally, NPs ended up accumulating in the liver, which is expected since it is known that the liver removes NPs from circulation, but each method of injection had a different profile. When administered orally, the MSN content in the liver decreased after the first 24 h, and most of the initial dose was excreted in faeces. For the intravenous route, the MSN content in the liver increased gradually up to 7 days after injection, presumably as NPs initially found in the lung, intestine, and muscle were subsequently discharged into circulation and phagocytosed into the liver and the spleen. The excreted NPs were found in urine, but curiously no damage to kidney tissue was apparent. Generally, the MSNs were determined to be biocompatible *in vivo* (Fu et al. 2013). Zhai et al. have shown that hollow MSNs can be degraded in human endothelial cells (Zhai et al. 2012).

In another study by Huang et al. longer MSNs (AR of 5) were taken up more by the spleen while the shorter variety (AR of 1.5) were taken up more by the liver (Huang et al. 2011). This trend was not observed with MSNs with ARs of 1, 1.75, and 5, when administered orally. In addition, oral administration did not result in excretion through the urine, unlike in the intravenous administration study (Li et al. 2015).

In addition to shape, surface modifications have been shown to have a great impact on biocompatibility, for example, poly(ethylene glycol) (PEG) grafting, or PEGylation, has been shown in one study to have a greater effect than on biocompatibility than aspect ratio (Huang et al. 2011). In this study, PEGylating led to increased lung accumulation, although a dissimilar result was seen in another study, which showed that PEGylating decreased lung accumulation (He et al. 2011). It has also been noted that PEGylated particles with a smaller size were more likely to escape capture from the RES (He et al. 2011). Overall, many studies agree that PEGylating increases blood circulation time and decreases excretion rate (Cauda et al. 2010; He et al. 2011), consistent with its known ability to delay opsonisation of the particles (Veronese and Pasut 2005).

Since a number of factors influence the behaviour of MSNs in biological systems, the biocompatibility of any particular MSN cannot be definitively stated, but in general MSNs are considered to be biocompatible. However, attempts at a more rigorous approach for the assessment of MSN biocompatibility are required, moving forward.

## MSN as a delivery vehicle for PDT agents

**Fig. 1:**
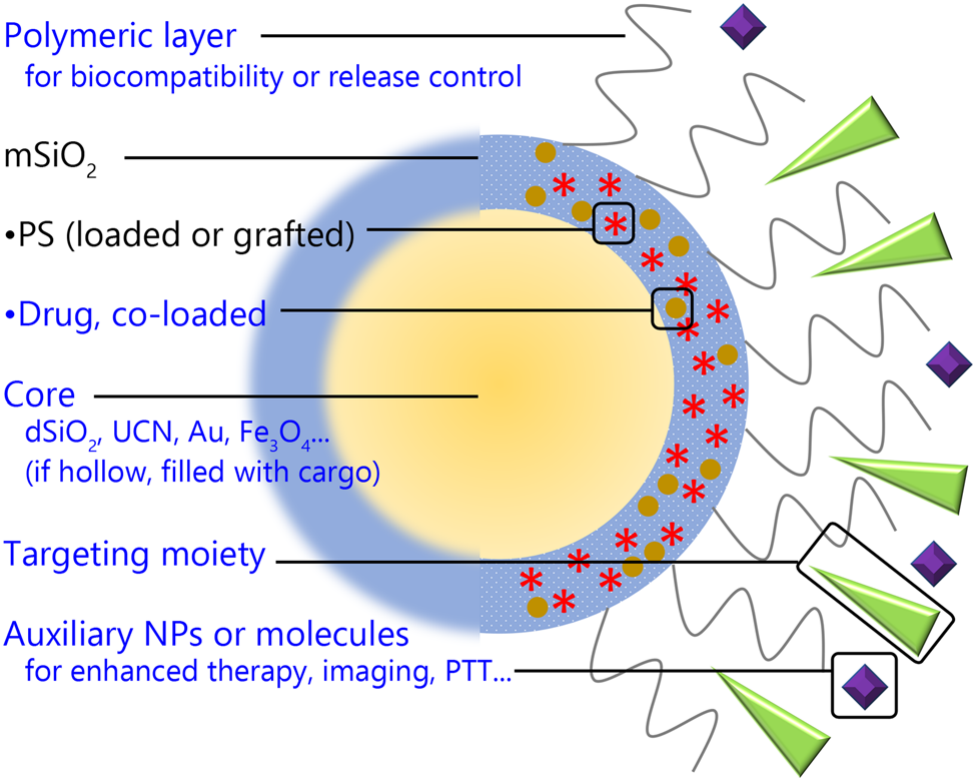
A general schematic drawing of a multifunctional PDT platform. All platforms have a mSiO_2_ base and a loaded PS. Optional features are indicated with blue text. Sometimes, a single component will serve multiple functions. (Color figure online)

The essential components for an MSN-based platform for PDT are mesoporous silica (mSiO_2_) and the PS molecule of choice, some of which are listed in Table 1. Many research groups have developed structures with more complexity, to allow for targeting, additional functions and altered biocompatibility, as shown in Fig. 1. A number of them incorporated other chemotherapy drugs such as doxorubicin (Dox) or cisplatin in the platforms as well, as it is known that PDT and chemotherapy in combination produce greater effects than a single therapy; and nanomaterials are an optimal platform to realize this synergy, as it allows local application of chemotherapy—which is traditionally a systemic treatment—through its targeting abilities, either by EPR or use of targeting moieties, and it combines two treatments into a single administration (Luo et al. 2017; Postiglione et al. 2011). In this section, we have classified studies based on the method of PS encapsulation inside the mSiO_2_, and whether an additional mechanism is built in to release cargo loaded inside the mSiO_2_ that can respond to environmental factors.

**Table 1:** List of recurring photosensitizers and their properties

Name	Abbreviation	Structure	Typical excitation wavelength	References
Hematoporphyrin	HP		630 nm	(Fan et al. 2014; Wen et al. 2016; Zhang et al. 2009)
*meso*-tetra(4-sulfonatophenyl)porphyrin	TPPS		645 nm	(Brevet et al. 2009; Gary-Bobo et al. 2012a; Gary-Bobo et al. 2012b) (derivative)
Protoporphyrin IX	PPIX		630 nm	(Qian et al. 2009b; Teng et al. 2013; Tu et al. 2009)
Chlorin e6	Ce6		660 nm	(Chai et al. 2017; Kamkaew et al. 2016; Su et al. 2017; Xu et al. 2017; Yang et al. 2017; Zhang et al. 2016)
Zinc phthalocyanine	ZnPc		680 nm	(Gnanasammandhan et al. 2016; Idris et al. 2012; Qian et al. 2009a; Tu et al. 2012; Wang et al. 2017)
Methylene blue	MB		666 nm	(Han et al. 2016; Planas et al. 2015; Wang et al. 2016)
Rose Bengal	RB		549 nm	(Hou et al. 2017; Zhan et al. 2017)
Merocyanine 540	MC540		556 nm	(Gnanasammandhan et al. 2016; Idris et al. 2012; Xu et al. 2017)
Indocyanine green	ICG		785 nm	(Guo et al. 2016; Hong et al. 2017b; Li et al. 2014)

## Platforms with simple PS loading

Various drugs have been loaded into mesoporous silica ever since ibuprofen was first loaded in 2001 (Vallet-Regi et al. 2001), PDT agents among them. In this type of drug-loaded nanoparticle, the primary goal is to move the cargo to the target cells efficiently. Qian et al. have synthesized MSN from organosilica within micelles, with the PDT agent protoporphyrin IX (PPIX) present (Qian et al. 2009b). They were then able to show ROS generation and qualitative phototoxicity in HeLa cells. Another group loaded silicon phthalocyanine dichloride (SiPcCl_2_) into MSN which was constructed using templating with cetyltrimethylammonium chloride (CTAC), the PS loading occurring in ethanol (Zhu et al. 2011). These researchers showed that SiPcCl_2_ encapsulated in the MSN was delivered to cells twice as efficiently as SiPcCl_2_ alone, and when phototoxicity was tested on HeLa cells, the SiPcCl_2_ MSN demonstrated up to seven times higher inhibition of cell proliferation. In a study published in 2012, a zinc phthalocyanine (ZnPc)-loaded MSN was developed, where layers of PEG and polyethylenimine (PEI) were used. Loading was performed using DMSO/ethanol as the solvent (Tu et al. 2012). Notably, the PEI layer took advantage of the ‘proton sponge’ effect that allowed the nanocomposite to escape efficiently from lysosomes into the cytosol. Mouse liver cancer cells (H22) were used to test the phototoxicity of the nanocomposite, which turned out to be higher than just the ZnPc-loaded MSN. Results from an in vivo model also showed efficacy and tumour specificity, which the authors attributed to the EPR effect.

**Fig. 2:**
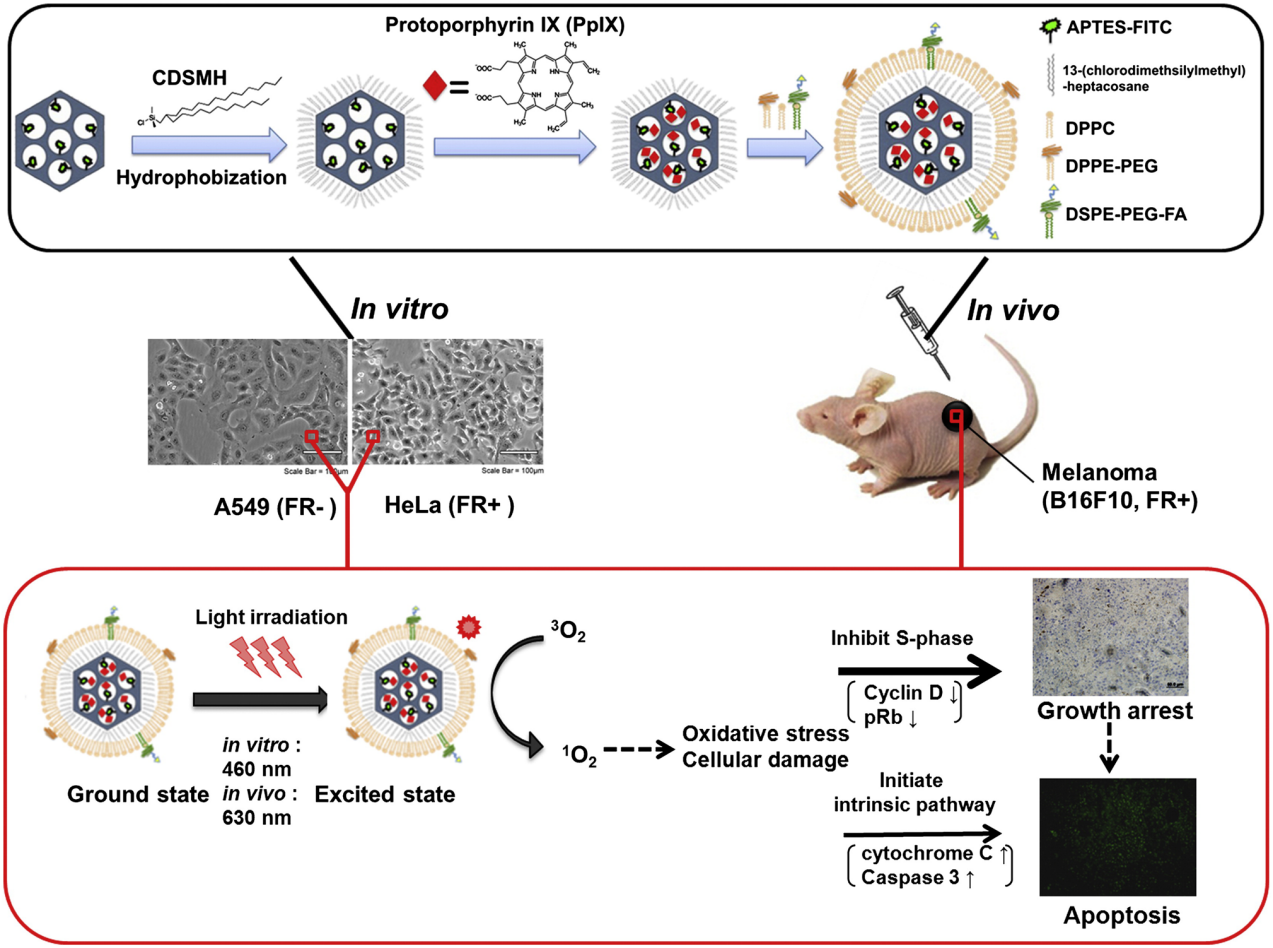
A representation of the design of PPIX-loaded MSN with FITC, lipid and folate, and the experiments conducted. Reprinted with permission from Teng et al. (2013) with permission from Elsevier

Yang et al. loaded the PS hypocrellin B to a peptide p160-conjugated MSN (Yang et al. 2014b). P160, a cancer-selective peptide, was attached to the aminated surface of the MSN using 1-ethyl-3-(3-dimethylaminopropyl)carbodiimide (EDC) chemistry, while the PS was loaded using dimethyl formamide (DMF). The authors showed that this loaded and targeted MSN was better internalized by cancer cells (MCF-7; human breast carcinoma cells) than by normal cells (ESF; human embryo fibroblast). Teng et al. loaded the PS, PPIX, into an MSN, capped it with phospholipid, and coupled it with folate (Teng et al. 2013) (Fig. 2). When tested with HeLa cells overexpressing the folic acid receptors (FAR) and A549 cells that have low FAR expression, the NPs were internalized more efficiently by the HeLa cells. Consequently, the phototoxic effect was high in HeLa cells but not in A549 cells. Compared with free PPIX, the NPs had a lower dark toxicity and a more efficient intracellular delivery. An in vivo study also showed efficacy in tumour volume reduction, and using immunostaining the authors elucidated the mechanism of tumour regression to be a combination of apoptosis and cell cycle arrest. Vivero-Escoto and Elnagheeb have demonstrated a simple approach of loading MSN with aluminium chloride phthalocyanine (AlClPc), a PS, and cisplatin, an alkylating drug (Vivero-Escoto and Elnagheeb 2015). The MSN was created by templating with CTAB, and drug loading was performed using DMSO. The resulting MSNs retained singlet oxygen generating ability, and were readily internalized by HeLa cells. The synergistic effect of AlClPc and cisplatin was shown using a phototoxicity experiment.

Zhang et al. loaded the hydrophobic PDT agent chlorin e6 (Ce6) into an MSN then coated its surface with cyclodextrin-grafted PEI, to which the cisplatin prodrug was covalently attached (Zhang et al. 2016). They reported, utilizing several methods, rapid internalization by endocytosis and release from endosomes through the aforementioned ‘proton sponge’ effect. In A549 cells, the combined therapy of PDT and chemotherapy had a higher cytotoxic effect than chemotherapy alone. As PDT has been used in antimicrobial applications, one group has also reported the use of MSNs in this area (Planas et al. 2015). In this study, methylene blue (MB) was loaded using ethanol, into a mannose-functionalized MSN. The dark toxicity of free MB for *E. coli* was abolished when encapsulated; overall, the phototoxicity of free MB and that of encapsulated MB were found to be virtually identical. Also, the effectiveness of mannose targeting was shown in *P. aeruginosa*.

MSN variations with different morphologies have also been used. A hollow type MSN (HMSN) has seen widespread usage due to the advantages discussed earlier. In a 2015 study, Ma et al. loaded 5-aminolevulinic acid (5-ALA), a PPIX precursor, into an HMSN synthesized by selective etching (Ma et al. 2015). The NPs were then conjugated with PEG and folic acid. The activity of the nanocomposite was then evaluated in B16F10 cells (skin cancer cells having a high level of FAR expression), and the improvement in phototoxicity over free 5-ALA was reported to be significant, especially in the low dosage range. Yang et al. have developed a fullerene (C_60_) loaded HMSN, co-loaded with Dox, an antitumour antibiotic (Yang et al. 2015). Synthesis was performed by condensing silica in an emulsion (in the presence of C_60_), then selectively etching out the solid core. They found that in MCF-7 cells, the combined chemo/phototherapy had higher toxicity than chemotherapy alone, which itself had similar toxicity to that of free Dox. An HMSN employing the exotic process of Cerenkov radiation has also been reported (Kamkaew et al. 2016). Here, the HMSN was loaded with the PDT agent Ce6, then its surface was chelated with ^89^Zr. This high-energy radionuclide decays through β^+^ emission, and the resulting luminescence matches the absorption spectra of Ce6. The PDT effect was subsequently demonstrated in both 4T1 mouse breast cancer cells and in vivo. Also positron emission tomography (PET) was successfully done in vivo, making use of ^89^Zr radiolabels.

**Fig. 3:**
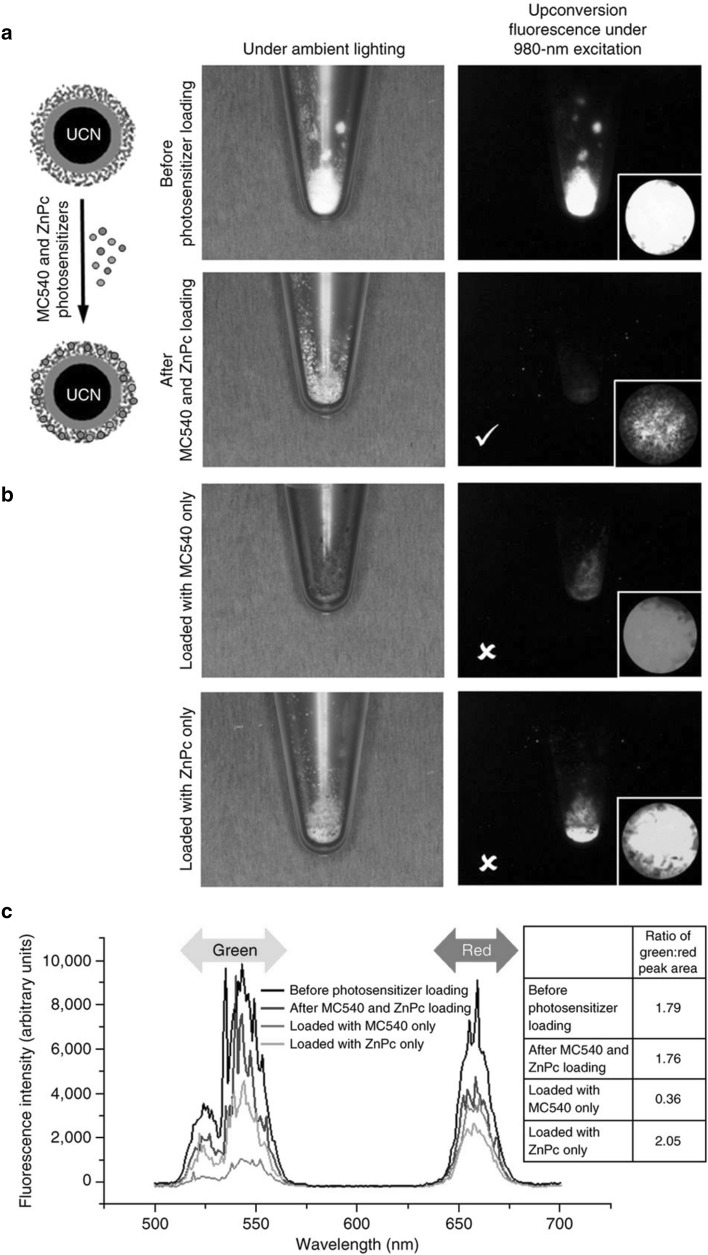
Physical appearances of UCN@mSiO_2_ under ambient lighting and 980 nm laser excitation **a** before and after dual loading with ZnPc and MC540, and **b** after loading with one of the PSs. The PSs absorb their respective wavelength ranges, as denoted in **c**, and luminescence that is not absorbed due to lack of PS in that wavelength range is observed in the picture. Reprinted by permission from Macmillan Publishers Ltd: Gnanasammandhan et al. (2016)

Some studies have combined other particles with mesoporous silica, although here we restrict our discussion to studies that that use silica as a drug carrier, rather than those that use silica simply as a coating layer. Upconversion nanoparticles (UCN) are a recurring theme in this area, as they allow the upconversion of incoming light, which means near-infrared (NIR) light can be converted to visible or UV light. This is naturally advantageous for PDT, because NIR light can be used for irradiation instead of visible light, which has poor tissue penetration (Liu et al. 2015). Two-photon excitation (TPE) has the same benefits, but its upconversion effect is less efficient than that of UCN and requires pulsed lasers, as opposed to the inexpensive continuous-wave lasers used for UCN (Gnanasammandhan et al. 2016). The hydrophobic nature of UCN requires a coating to make them more hydrophilic and modifiable, which can be fulfilled using a mesoporous silica layer (Liu et al. 2015). A study reported in 2009, made use of a UCN made of NaYF_4_:Yb,Er which was coated with dense silica and then with mesoporous silica (created by calcining octadecyltrimethoxysilane, C_18_TMS), after which ZnPc was loaded into the nanoparticle using pyridine. The imaging and SOG performances of the nano-composite were then evaluated to be effective. Idris et al. covered a NaYF_4_:Yb,Er UCN with mesoporous silica. Remarkably, two PSs were loaded into the mesoporous layer: this type of UCN typically has an emission spectra with two peaks at green and red upon NIR (980 nm) irradiation, therefore merocyanine 540 (MC540) was loaded to utilize green range and ZnPc for red range (Idris et al. 2012) (Fig. 3). The product was shown to be more effective in PDT in B16-F0 mouse melanoma cells than in cells loaded with only one of these PSs. Furthermore, in vivo experiments showed PDT therapeutic efficacy when conjugated with folic acid and PEG. A protocol published in 2016 provides the detailed synthesis of a NaYF_4_:Yb,Er(or Tm) UCN covered in amorphous silica then mesoporous silica, with MC540 and ZnPc as cargo, and also functionalized with PEG or folic acid (Gnanasammandhan et al. 2016).

Wang et al. created a multimodal complex consisting of UCN, mSiO_2_ and CuS NPs (Wang et al. 2017). First, NaGdF_4_:Yb,Er,Mn,Co UCN was coated with a mesoporous silica layer (using CTAB templating), and then ultra-small CuS NPs were deposited on the surface, following this ZnPc and Dox were loaded (in DMSO and buffered saline, respectively) into the mesoporous silica layer. UCN doping with Mn^2+^ increases the red light ratio of emitted light which will be picked up by ZnPc; the presence of CuS NPs allows for use in photothermal therapy (PTT). The effect of this multimodal complex combined therapy was confirmed using HeLa cells, demonstrating that the effects of the individual treatments were additive; similar data were also obtained *in vivo*. In addition, computed tomography (CT)/magnetic resonance (MR) imaging capabilities, provided by the doped UCN, were also demonstrated.

Gold nanorods (AuNR) have also been used in conjunction with mesoporous silica. A study by Li et al. used an MSN enclosing AuNR the surface plasmon resonance of which was tuned to match the absorption of ICG loaded in the silica layer (Li et al. 2014). This promoted the absorption of light by ICG and PDT efficacy, as shown by treatment of MDA-MB-231 human breast cancer cells and in vivo.

Iron oxide is another example of a material that can be incorporated into an MSN. In one study, Guo et al. started with magnetite (Fe_3_O_4_), coated it with polydopamine, and then mesoporous silica. PEG chains were then attached and a lipophilic triphenylphosphonium cation (for endosomal escape and mitochondrial targeting) and transferrin (for tumour cell uptake) were also conjugated (Guo et al. 2016). indocyanine green (ICG) was then loaded to allow for both PDT and PTT. The authors showed effective phototherapy efficacy in vitro using A549 cells, and furthermore, in vivo studies demonstrated therapeutic efficacy of phototherapy, bio-distribution of the NPs (predominant in liver and spleen, with no significant systemic damage), and MR imaging capability from the magnetite core.

## Platforms activated upon internalization

**Fig. 4:**
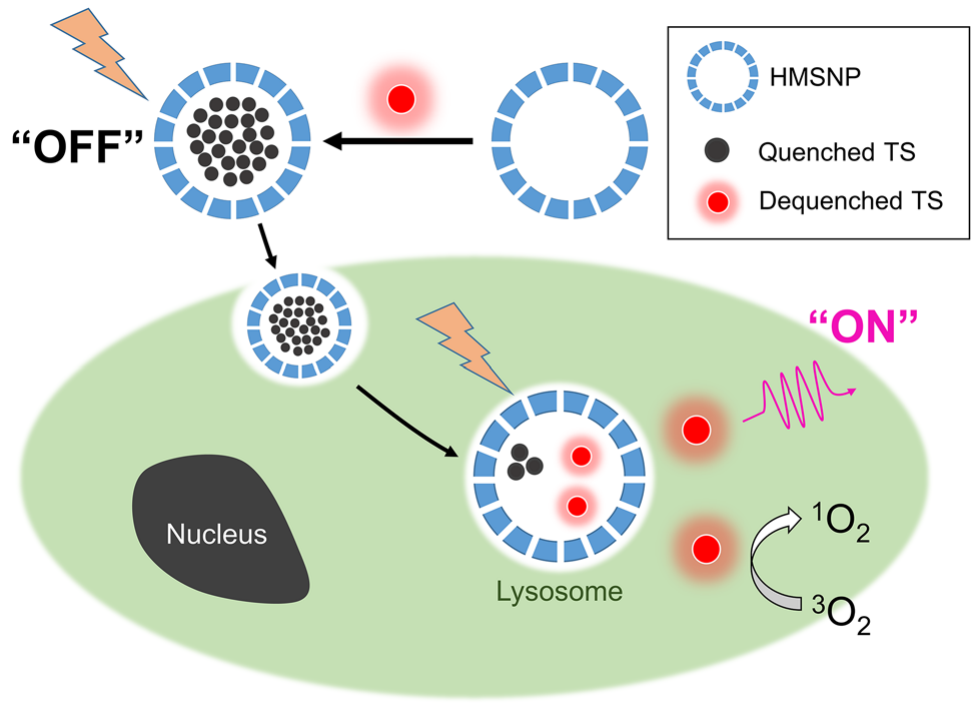
A schematic drawing of the ‘off–on’ property of talaporfin-loaded HMSN. TS stands for talaporfin sodium. When particles are internalized by cancer cells, photosensitizer is released and becomes highly fluorescent and phototoxic. Reprinted with permission from Hong et al. (2017a)

We have recently showed for the first time that ‘off–on’ type of PDT agents could be developed by loading PS inside HMSN so that the physical properties of PS are suppressed. In one paper, a hydrophilic PS talaporfin was loaded, via aqueous solution, in HMSN (created from hard templating with polystyrene beads) and it was capped with PEG (Hong et al. 2017a). The talaporfin molecules were initially confined to small spaces when adsorbed onto the HMSN, but after cell internalization, they became disaggregated. At first, through fluorescence resonance energy transfer (FRET), their SOG and fluorescence are largely quenched. Once internalized, their fluorescence and photodynamic capacity were restored, and this recovery was shown through both fluorescence and flow cytometry. This is a novel property which we refer to as ‘off–on’, meaning the system remains inactive (both fluorescence and SOG) while outside the cells (Fig. 4). This could potentially solve problems rising from nonspecificity, including poor imaging contrast and skin photosensitivity. Also, because free talaporfin molecules, being hydrophilic, are difficult to transport across cell membranes, cell uptake greatly increased after encapsulation. Overall, the talaporfin-loaded HMSNs showed a greater phototoxicity than free talaporfin when used to treat the mouse squamous cell carcinoma SCC-7. In another study, ICG was loaded into HMSNs (Hong et al. 2017b). ICG is relatively unstable and disintegrates in aqueous solution, a process that is accelerated by heat and irradiation; in addition when intravenously injected ICG shows nonspecific plasma binding, and is quickly cleared from circulation. By using encapsulation, these issues were addressed, and the composite had a similar ‘off–on’ property as was observed for talaporfin, demonstrating an improved phototoxic effect on SCC-7 cells. The ‘off–on’ effect also extended to the photothermal effect of ICG, which was also suppressed when ICG aggregated. These data showed that MSN platforms can be developed to have smart functionalities without the need for a complex design.

## Platforms using covalent attachment of PS

It is possible to covalently attach PS molecules to the surface of nanoparticles. However, relatively few researchers utilize this method, possibly because the excellence of MSN as a drug carrier, as discussed above, does not necessitate covalent bonding for successful incorporation. One natural consequence of grafting is that PS molecules will not leave the nanocontainer under normal circumstances (Couleaud et al. 2010; Fan et al. 2014). In a study reported in 2009, Tu et al. reported covalent conjugation of PPIX molecules to an MSN to examine the possibility of using MSN in PDT (Tu et al. 2009). This was performed by functionalizing the silica surface with amine groups and then conjugating them with succinimide-modified PPIX. As a result, self-quenching did not occur, and the absorption spectra before and after encapsulation were similar to each other. The PPIX-conjugated MSN was effectively taken up by cells, and showed dose- and time-dependent phototoxicity in HeLa cells. In another study, Brevet et al. conjugated TPPS-derived PS, by synthesizing MSN with TEOS and silane modified with PS, using mannose as a targeting moiety for the MSN matrix (Brevet et al. 2009). MDA-MB-231 cells were used as the target cells, and mannose receptors were shown to play a role in endocytosis of the targeted MSN.

**Fig. 5:**
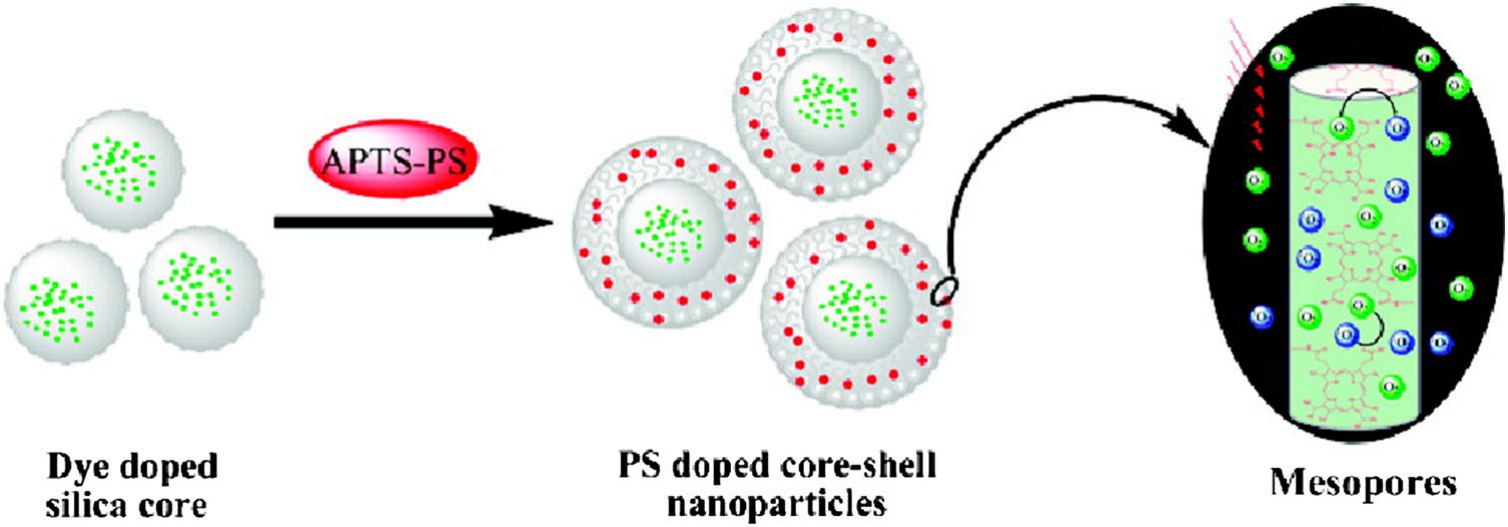
Procedure of synthesis for dSiO_2_@mSiO_2_ with HP-conjugated mesopores. Reprinted with permission from Zhang et al. (2009). Copyright 2009 American Chemical Society

In addition, in a study published in 2009, Zhang et al. presented a nanocomposite system constructed by coating a nonporous, FITC-doped silica core, with a mesoporous silica layer conjugated to hematoporphyrin (HP) also using APTES (Zhang et al. 2009) (Fig. 5). These researchers showed that at the same HP equivalence, the nanocomposite produced more singlet oxygen than HP alone, because of its greater stability inside the silica shell. Zhao et al. have also conjugated tetrasubstituted carboxyl aluminium phthalocyanine (AlC_4_Pc) to the surface of a hollow MSN, after which the particle was coated with Pd nanosheets (Zhao et al. 2014). Because AlC_4_Pc and Pd nanosheets have a similar absorption range, they were able to perform both PDT and PTT at the same time. The uptake and phototoxicity of this nanocomposite was tested on HeLa cells.

In another study, Gary-Bobo et al. grafted tri(4-sulfatophenyl)porphyrin into an MSN and loaded camptothecin, a chemotherapy drug, and covered the particles with galactose (Gary-Bobo et al. 2012b). Phototoxicity was confirmed using MDA-MB-231 and HCT-116 human colorectal cancer cells. In related research, an MSN grafted with the same PS was coated with poly-(L-lysine) and hyaluronic acid (HA), which targets CD44 receptors (Gary-Bobo et al. 2012a). The researchers found no dark toxicity of this agent, and confirmed the role of HA in its uptake by showing that its phototoxicity decreased in HCT-116 cells if they were pre-incubated with HA. Another group covalently attached a PS verteporfin to an MSN framework embedded with Rhodamine B (Rizzi et al. 2017). The conjugated platform was shown to possess no inhibitive effects to normal human keratinocyte HaCaT cells or a poorly metastatic melanoma cells A375P cells, but the platform had dramatic effects on highly invasive SK-MEL-28 melanoma cells. Specifically, when treated with the platform, SK-MEL-28 proliferation was reduced and their morphology was drastically deformed. When the cells were treated with endocytosis inhibitors, those effects were attenuated, suggesting that the platform functions through an internalization dependent mechanism.

Some studies involving UCNs have also implemented covalent attachment, though only partially. Fan et al. used a Gd^3+^ doped UCN, which can function as an MR contrast agent, and created a rattle structure by coating it with dense silica and then mesoporous silica, after which the former was selectively etched away. A PS, HP, was then attached covalently, and the chemotherapy drug docetaxel (Dtxl) was loaded, both of which also possess radiosensitising ability. A synergistic chemo-/radio-/photodynamic therapy was demonstrated both in vitro (HeLa cells) and in vivo (Fan et al. 2014). Xu et al. used a UCN, tuned to have a more biocompatible absorption through its core–shell structure (NaGdF_4_:Yb,Er@NaGdF_4_:Nd,Yb), that was additionally sensitized with the IR-808 dye as an antenna, and coated it with a mesoporous silica layer which had Ce6 covalently attached for red emission and loaded with MC540 for green emission. The therapeutic efficacy of this nano-composite was demonstrated in HeLa cells and in vivo (Xu et al. 2017).

Croissant et al. have reported the use of TPE PDT with an MSN, by covalently attaching a two-photon PS to the pores of the MSN, and then also adding gold NPs to enhance the efficiency of the system (Croissant et al. 2016). These researchers showed that both the gold and the two-photon PS improved the treatment efficacy in MCF-7 cells. These workers also demonstrated TPE imaging in vitro using this nano-composite.

## Platforms for externally triggered therapy

Various techniques to gate the release of cargo from MSN have been introduced, including mechanically controlling polymer molecules that block the pores, or introducing a cleavable chemical bond (Kwon et al. 2013; Li et al. 2012). Here, studies that have introduced such a method to explicitly control the release of cargo are discussed; nevertheless, it should be noted that some of the simpler approaches outlined in the previous sections also provide some form of control over cargo release, for example through well-known (Hong et al. 2017b; Ma et al. 2015) pH-dependent electrostatic interactions between surface functional groups such as amine and cargo molecules (Planas et al. 2015; Wang et al. 2017; Yang et al. 2015) or with assistance from photothermal effects (Li et al. 2014; Wang et al. 2017). In the following studies, the externally controlled release is generally limited to the co-loaded drug, and the PS molecules are either covalently attached or their release profiles have not been explored.

**Fig. 6:**
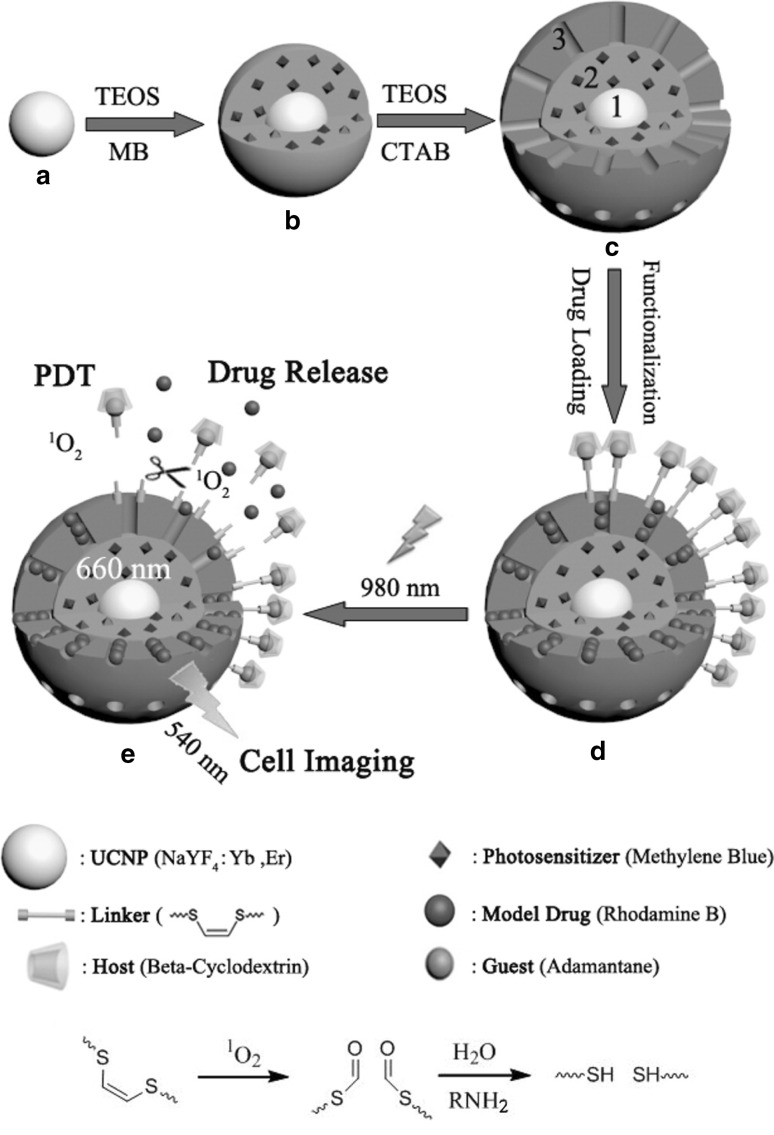
Synthesis process of UCN@dSiO_2_(-MB)@mSiO_2_ loaded with Rhodamine B and capped. Chemical mechanism for the singlet oxygen-responsive linker. Reprinted with permission from Wang et al. (2016). Copyright 2016 American Chemical Society

Yang et al. coated UCN@mSiO_2_ with the copolymer PEG-*b*-MAPS, in which the 9,10-dialkoxyanthracene groups convert upon reaction with singlet oxygen to 9,10-anthraquinone resulting in polymer degradation (Yang et al. 2014a). Ce6, covalently attached to the MSN shell, served both as a PDT agent, and also as a light-responsive trigger that released loaded drug (Dox) by converting irradiation (via an upconversion process) into breakdown of the polymer envelope. These workers demonstrated the release response and then the synergistic effect between PDT and Dox in vitro in KB cells and in vivo. In a similar manner, Lv et al. coated UCN@mSiO_2_ with poly(N-isopropyl acrylamide)-(methacrylic acid), which is both temperature and pH sensitive (Lv et al. 2015). Following this, Au_25_(SR)_18_ clusters were loaded onto the mesoporous layer, along with Dox. When irradiated, the upconverted luminescence induced PDT and PTT from the gold cluster, and the increase in temperature led to the degradation of the polymer layer and Dox release. At a lower pH the release was accelerated, which offers a potential benefit because the tumour environment is typically acidic. This triple therapy was tested in both A549 cells and in vivo. Wang et al. used a triple layer design of mesoporous silica over solid silica over UCN, where the PS (MB) was embedded in the solid silica, and the model drug Rhodamine B was loaded into the mesoporous layer (Wang et al. 2016). Then, in order to block the pores, beta-cyclodextrin was introduced to the surface, linked by a singlet oxygen sensitive linker connected to 1-adamantane. The PDT effect was demonstrated in A549 cells (Fig. 6). Another group used a similar construction of three layers, using MB as the PS and Dox as the drug (Han et al. 2016). Here, the polymer coating used was PEI-folic acid; this layer dissociated from the particle at low pH, prompting the release of Dox. These studies reported on the release profile and SOG.

Tong et al. augmented UCN@mSiO_2_ by using a UV-cleavable linker (an o-nitrobenzene derivative) inside the pores; in addition, TiO_2_ was synthesized inside the mesoporous layer as a PDT agent, Dox was loaded into the nanoparticle, and the surface was modified with folate for targeting (Tong et al. 2017). Upon NIR irradiation, the UCN emits UV light which breaks the linker and releases Dox. The study examined the responsive release profiles and reported on the therapeutic effects in HeLa cells. Hou et al. created a system also based on UCN@mSiO_2_. First, the PS, Rose Bengal (RB), was grafted onto the silica, followed by azobenzene groups (Hou et al. 2017). Dox was then loaded as the drug. Once irradiated, the UCN emits both green light and UV light: the green light is absorbed by RB for PDT, and the UV light by the azobenzene groups: upon absorbing light energy the azobenzene groups move mechanically, and through such ‘wagging’, the release of Dox molecules is promoted. This series of events was supported by release experiments and the anti-tumour effect was tested on HeLa cells.

Aside from UCN, iron oxide, including superparamagnetic iron oxide (SPIO), has also been used in conjunction with a gated MSN. For example, Zhan et al. created Fe_3_O_4_ NP wrapped with a mesoporous silica layer over a solid silica layer, and this was loaded with RB (Zhan et al. 2017). A pH-responsive copolymer of PEG and poly(aspartic acid), PEG-*b*-PAsp, was then coated around it. This layer was shown to increase the loading capacity and allowed for pH-responsive release. Improved therapeutic effects were demonstrated against B16 mouse melanoma cells and furthermore, in vivo, the possibility of magnetic targeting was also demonstrated as well as effective anti-tumour performance. In another study, Yang et al. started with an Fe_3_O_4_ NP, grew ultrafine gold nanocrystals on its surface, and coated it with mesoporous silica. Dox and Ce6 were then loaded into this nanoparticle, polyelectrolyte multilayers of alginate/chitosan were deposited and a P-gp small hairpin RNA (shRNA) was then attached (Yang et al. 2017). In this complex system, the SPIO allows for MR imaging and magnetic targeting, gold is used for computed tomography (CT) imaging, the multilayers act as gatekeepers sensitive to pH, and the P-gp shRNA interferes with multiple drug resistance by down-regulating P-glycoprotein. In this work, notably the pH-responsive release of both Dox and Ce6 was shown: Dox was released more in pH 4.0 than it was in pH 7.4 and Ce6 was released more in pH 7.4 than it was in pH 4.0. The NPs were shown to be nontoxic in the absence of light, and also haemocompatible. Cellular uptake was shown through imaging, with the NPs mainly being located in the lysosomes. Magnetic targeting was shown, as more NPs were taken up by cells closer to a magnet. The synergy between the chemotherapy and PDT combinations was tested using MCF-7 cells, mouse breast cancer EMT-6 cells, and adriamycin resistant MCF-7/ADR cells, and the effects were all significant, especially with the Dox-resistant MCF-7/ADR cells. In vivo, anti-tumour effects against EMT-6 cells were also demonstrated along with MR imaging capability. MR and CT imaging were also performed in vitro.

Other studies have used MSNs only. Chai et al. have reported a cyclodextrin-gated MSN containing Ce6. In this system, the Ce6 and the model cargo (either Rhodamine B or calcein) were loaded, and the cyclodextrin molecule was linked to the surface using azobenzene and a singlet oxygen-sensitive linker (Chai et al. 2017). Red light irradiation resulted in SOG from Ce6, which acts as a source for PDT, but also cleaved the linker and opened the pores by removing the cyclodextrin gates.

Wen et al. have reported on the use of an HP-conjugated MSN loaded with Dox, and coated with CeO_2_ (ceria) (Wen et al. 2016). Ceria NPs are known to decompose into cerium ions in the presence of a reductive environment. These authors showed the release of Dox following the dissolution of the ceria layer triggered by glutathione (GSH) treatment, which was facilitated further with irradiation and a low pH. In cytotoxicity experiments, the material showed toxicity comparable to free Dox against HeLa cells, but was significantly less toxic than free Dox against 293T human embryonic kidney cells, thereby showing specificity. In another example, MSN dual-loaded with Ce6 and Dox (in phosphate buffered saline) was covered in a red blood cell membrane, so that the particles would be stable in circulation and evade possible attacks from the immune system (Su et al. 2017). Once the particles were irradiated, the SOG by Ce6 destroyed the erythrocyte membrane and released the Dox. Enhanced delivery and light-triggered release were all verified in this study, and the dual therapy was tested on 4T1 cells both in vitro and in vivo. Furthermore, in vivo, improved tumour-specific uptake (likely due to an increased EPR effect arising from longer circulation) and its ability to suppress metastasis were also demonstrated.

## Conclusion

Mesoporous silica nanoparticles, having been extensively researched as drug carriers, can be used for PDT with the addition of various functions. In this review, we focused on recent advances in photosensitizer delivery systems that involve mesoporous silica as the main functional component. Most importantly, photosensitizers and, optionally, other drugs were loaded or grafted onto the mesoporous silica. Mesoporous silica’s ease of functionalization allows the attachment of targeting moieties, responsive polymers, or other auxiliary nanoparticles. In many of the systems introduced, using MSN increases uptake efficiency, consolidates multiple functions into one theranostic platform, improves cargo stability, ensures biocompatibility, reduces unwanted exposure of normal tissues to therapy, and improves the treatment effect through synergy between the different components. Numerous in vitro and in vivo therapeutic effects have been described in the literature which was extensively reviewed here.

In the future, activatable platforms to enhance the specificity of treatment and reduce the systemic toxicity will continue to grow in importance. Out of the numerous emergent techniques for drug delivery that use mesoporous silica, there remains a significant amount to be implemented in the field of PDT. Also, more platforms need to be made multifunctional—for example, they can be supplemented by a second mode of therapy, or imaging abilities can be added to the drug delivery systems, as several number of imaging methods have been explored already as seen in this review: fluorescence (including upconversion luminescence and two-photon imaging), MR, CT and PET. MSN is a therefore a prominent and evolving carrier that can be used to supplement traditional PDT.
